# Note on a New Antipruritic Remedy

**Published:** 1882-02

**Authors:** 


### NOTE ON A NEW ANTIPRURITIC REMEDY.

Dr. Bulkley has directed attention to a very important point which is often a source of great anxiety to the practitioner, viz.: the difficulty in relieving persistent and wearing itching in skin affections. He points out that the drugs we certainly rely on, viz.: opium, morphia, chloral, bromide of potassium, aconite, and carbolic acid, when administered internally, often fail to stop the unconscious scratching, and he was led from the known effects of gelseminum to try that drug. In certain cases he has found it decidedly efficacious. He begins with ten drops of the tincture, and, if in half an hour there is no relief, he gives twelve or fifteen drops, and so on, until one or two drachms have been reached in two hours.—*N. Y. Med. Journ.*

